# *Serratia odorifera* a Midgut Inhabitant of *Aedes aegypti* Mosquito Enhances Its Susceptibility to Dengue-2 Virus

**DOI:** 10.1371/journal.pone.0040401

**Published:** 2012-07-27

**Authors:** Anjali Apte-Deshpande, Mandar Paingankar, Mangesh D. Gokhale, Dileep N. Deobagkar

**Affiliations:** 1 Molecular Biology Research Laboratory, Department of Zoology, Center for Advance Studies, University of Pune, Pune, India; 2 Department of Medical Entomology and Zoology, National Institute of Virology, Pune, India; 3 Vice Chancellor, Goa University, Taleigaon Plateau, Goa, India; University of Texas Medical Branch, United States of America

## Abstract

Mosquito midgut plays a crucial role in its vector susceptibility and pathogen interaction. Identification of the sustainable microflora of the midgut environment can therefore help in evaluating its contribution in mosquito-pathogen interaction and in turn vector competence. To understand the bacterial diversity in the midgut of *Aedes aegypti* mosquitoes, we conducted a screening study of the gut microbes of these mosquitoes which were either collected from fields or reared in the laboratory “culture-dependent” approach. This work demonstrated that the microbial flora of larvae and adult *Ae. aegypti* midgut is complex and is dominated by Gram negative proteobacteria. *Serratia odorifera* was found to be stably associated in the midguts of field collected and laboratory reared larvae and adult females. The potential influence of this sustainable *g*ut microbe on DENV-2 susceptibility of this vector was evaluated by co-feeding *S. odorifera* with DENV-2 to adult *Ae. aegypti* females (free of gut flora). The observations revealed that the viral susceptibility of these *Aedes* females enhanced significantly as compared to solely dengue-2 fed and another gut inhabitant, *Microbacterium oxydans* co-fed females. Based on the results of this study we proposed that the enhancement in the DENV-2 susceptibility of *Ae. aegypti* females was due to blocking of prohibitin molecule present on the midgut surface of these females by the polypeptide of gut inhabitant *S. odorifera.*

## Introduction

The gut flora of the vertebrates and invertebrates represents one of the most widespread and ancient symbiotic association. This symbiosis provides important physiological functions to the host, including the synthesis of essential nutrients, resistance to colonizing pathogens and stimulation of immune system [1], [2]. In hematophagous arthropods, these symbionts are even thought to be critical for the host fitness because of the need for blood scarce nutrients.

The mosquito midgut is an immune competent organ and is the first point of contact between the vector and arboviruses. An understanding of the microbial community structure of the mosquito midgut will therefore be crucial to identify its role in virus entry, multiplication and in turn, vector susceptibility. Recent studies of the mosquito midgut have utilized both culture dependent and independent methods to detect diverse microbial community in this physiological niche [2], [3], [4], [5], [6], [7], [8], [9], [10], [11], [12], [13], [14], [15] (also see Table S1). These studies mainly focus on the bacterial diversity and prevalence of certain bacterial species in the midguts of different mosquitoes like Anopheles, Aedes and Culex. Some of these reports also discuss the change in the midgut microbiota during developmental stages of mosquitoes [12], [14]. A Recent report has shown that in-vitro interaction of La Crosse virus with the midgut bacteria, isolated from *Ae. albopictus*, significantly reduces the infectivity of La Crosse virus (transmitted by this vector) to Vero cells [15]. This study warrants further investigation of this phenomenon in living mosquitoes. Similarly, Cirimotich et al [13] have demonstrated refractoriness to Plasmodium infection due to the generation of reactive oxygen species by resident gut microbes. Barring few of these examples, not much is understood about the mechanism underlying the altered arboviral or parasite susceptibility due to presence of gut microbes in these vectors.

*Aedes aegypti* is a natural vector for a group of viruses such as chikungunya, dengue fever virus, yellow fever virus as well as protozoan parasite, *Plasmodium gallinaceum*. It has been demonstrated that strains of *Ae. aegypti* also support the complete development of the filarial worms *Brugia malayi* [16], *Brugia pahangi* [17], and *Dirofilaria immitis* [18]. In spite of its epidemiological importance in disease transmission, very limited studies are available on *Ae. aegypti* with respect to the identification of gut microflora and its interaction with the disease transmitting agent. Identification of the stable and transmissible gut inhabitant of such a vector would therefore be a crucial factor in understanding its susceptibility to battery of viruses and parasites.

We employed culture dependent approach to investigate the bacterial diversity of *Ae. aegypti* midgut. A screening study of the midgut bacteria of *Ae. aegypti* mosquitoes reared in the laboratory and collected from the field was carried out to identify the microbes that are stably present in the midgut of these mosquitoes. The status of the midgut microbial community was evaluated during developmental stages of these mosquitoes by collecting larval stage from different locations and allowing them to metamorphose in laboratory. Additionally, to understand the impact of higher ambient temperature on the gut microbes, larval population was collected from the fields during extreme summers and in the laboratory, similar condition was mimicked by exposing the larvae to higher temperature prior to their dissection.

Further studies were carried out to understand the role of the selected gut microbes in DENV-2 susceptibility of *Ae. aegypti* by artificially introducing them in the mosquito midgut (free of gut flora) through blood meal. Additionally, we also analyzed the interaction among the selected gut inhabitant and gut epithelial polypeptides in-vitro and in-vivo. Based on the observations of this study, we have proposed the underlying mechanism responsible for the enhanced DENV-2 susceptibility of these mosquitoes in the presence of the specific gut microbe.

## Results

### Culturable Microbiota from the Midgut of *Ae. aegypti*

Our studies identified, total 13 bacterial isolates from the midguts of field collected and laboratory reared *Ae. aegypti* by traditional biochemical methods and 16s rDNA sequence analysis (Table 1). Based on the 16s rDNA sequence analysis, the isolated midgut microbes were classified into three major groups, viz., gamma-proteobacteria, beta-proteobacteria and fermicutes. Biochemical characterization based identification of the isolates corroborated with their 16s rDNA sequence based identification. The sequences obtained in this work are deposited in Genbank database (Table 1).

**Table 1:** Relative percent distribution of microbes in fourth *in star* larval midgut and their surrounding habitat.

	Field Collection						Laboratory Collection					
Microbe*	Pune		Pune summer		Ahamedabad		AFMC		MCC		NCL	
	Water	Midgut	Water	Midgut	Water	Midgut	Water	Midgut	Water	Midgut	Water	Midgut
*Aeromonas hydrophila* (DQ855289)	−	−	−	−	−	−	−	−	14.82	35.45	−	−
*Aeromonas media* (DQ855288)	−	−	10.95	42.48	10.71	64.29	Present	54.67	−	−	−	−
*Aeromonas salmonicida smithia* (DQ855287)	8.07	39.06	−	−	−	−	−	−	−	−	9.64	44.75
*Bacillus cereus* (DQ855295)	−	−	1.50	1.77	1.79	−	−	−	−	−	−	−
*Brevibacillus agri* (DQ855294)	−	−	−	−	3.57	2.23	−	−	−	−	−	−
*Edwardsiella tarda* (DQ 855293)	5.60	6.15	−	−	−	−	−	−	−	−	−	
*Pantoea agglomerans* (DQ 855292)	33.94	32.94	15.30	11.77					−	6.05	−	−
*Enterobacter cloacae*	−	−	−	−	−	−	−	3.53	−	−	−	−
*Enterobacter gergoviae*	−	−	−	−	−	−	−	−	−	−	22.11	−
*Escherechia coli*	11.04	−	3.85	−			−	−	−	−	−	−
*Microbacterium laevaniformans*	14.09	−	−	−	−	−					−	−
*Microbacterium oxydans* (DQ294032)	−	−	−	−	−	−	Present	21.63	6.93	8.50	7.29	4.23
*Providencia rustigianii*	4.61	−	−	−	−	−	−	−	−	−	−	−
*Pseudomonas alcaligenes* (DQ855290)	2.80	−	55.47	10.35	23.21	10.21	−	−	−	−	38.58	18.52
*Burkholderia mallei* (DQ855291)	3.95	4.08	−	−	−	−	Present	13.27	−	−		
*Ralstonia pickettii*	−	−	−	−	−	−	−	−	−	−	12.75	−
*Burkholderia pseudomallei*	−	−	−	−	−	−	−	−	3.45	−	−	−
*Pseudomonas putida* (DQ294033)	−	8.37	−	−	−	−	−	−	37.50	44.57	−	−
*Serrattia odorifera biogroup I* (AY859722)	9.23	−	−	−	−	−	−	−	−	−	−	−
*Serrattia odorifera biogroup II*	2.14	9.40	2.08	33.63	30.33	23.27	Present	6.88	38.75	5.43	7.52	32.50
*Xenorhybdus luminiscens*	4.53	−	10.90	−	30.36	−	−	−	−	−	−	−
Unidentified	−	−	−	−	−	−	−	−	−	−	2.11	−

* Identification of isolates is based on conventional biochemical characterization and 16s rDNA sequencing.

% Relative distribution = CFU of a particular type of microbe/CFU of all types of microbes in given sample/niche ×100.

A large proportion of the isolates (64%) were identified as Gamma-proteobacteria, where dominant genera were *Aeromonas, Enterobacter and Pseudomonas.* For the mosquitoes reared in three different laboratories, we observed existence of ten different microbes in their midguts with *S. odorifera* biogroup II and *Microbacterium oxydans* as common gut inhabitants (Table 1). Additionally, in the field collected mosquitoes, 11 distinct isolates were identified with a dominance of *Aeromonas media*, *Serratia odorifera* biogroup II and *Pseudomonas alcaligenes* (Table 1). *S. odorifera* was the common gut microbe of all the mosquitoes analyzed in this study.

To know the source of these gut inhabitants, the bacterial consortium of the respective larval habitat was also analyzed. Our observations suggested that the larval surroundings influenced the type of gut microbes they harbored (Table 1). Conversely, it was also observed that the microbes like *Xenorhybdus luminiscens* and *Escherichia coli;* although present in abundance in the larval habitats, did not colonize their guts suggesting selective retention of specific microbes as midgut inhabitants (Table 1).

### Transstadial Transmission

The gut microbial community was monitored during metamorphosis of mosquitoes to investigate the dynamics of this community during the transition from larvae to adult. *S*. *odorifera* was the only microbe commonly associated in the midguts of pupae, emerging imagoes and adults of all field collected and laboratory reared *Ae. aegypti* (Table 2). These observations suggested that *S. odorifera* was transstadially transmitted from larvae to adult.

**Table 2:** Culturable microbiota present in the larval habitat and laboratory reared/field collected *Ae. aegypti* mosquito midguts during developmental stages.

		Field Collection			Laboratory Collection		
		Pune	Pune summer	Ahemadabad	AFMC	MCC	NCL
1	No. of bacterial species in the larval midguts	6	5	4	5	5	4
2	No. of bacterial species in the midguts of pupae/imagoes*	1	1	1	1	1	1
3	No. of bacterial species in the larval midguts after heat shock**	2	1	1	2	2	2
4	No. of bacterial species in the water from larval collection site	11	7	6	3	5	7
5	Total mean CFU load/midgut (SD)	3.84×10^4^ (0.58×10^4^)	2.4×10^4^ (0.4×10^4^)	4.04×10^4^ (0.11×10^4^)	2.01×10^4^ (0.75×10^4^)	3.46×10^4^(0.12×10^4^)	2.26×10^4^(0.29×10^4^)

* *S. odorifera* was found in the midguts of pupae and imagoes of all *Ae. aegypti* collected from fields and laboratories.

** Along with *S. odorifera*, *M. oxydans* also sustained in the larval midguts after heat shock treatment.

### Effect of Heat Shock Treatment on the Bacterial Composition in the Fourth Instar Larval Gut

During our study, it was observed that the status of the midgut flora changed significantly during the developmental stages of this vector as it transits from aquatic to terrestrial life. It was therefore important to understand if the higher ambient temperatures can affect the dynamics of gut microbiota in larval stage which leads mainly aquatic life and is predominantly exposed to severe temperatures than other stages. The ambient temperature in summer season in Pune and many other cities in India reaches up to 41°C or more, hence, to simulate this condition in the laboratory, a heat shock exposure (41°C) was given to fourth instar larvae and the survivors in the gut were analyzed. Consequently, the larvae were also collected from the natural habitats during summer season when ambient temperature was 39°C.

There was a significant reduction of about two logs in CFU of gut microbes after high temperature exposure of the fourth instar larvae. The average CFU of the gut microbes after heat shock exposure of larvae collected from AFMC showed a reduction from 2×10^4^ to 5×10^2^ while it decreased to 4.8×10^2^ from initial count of 3×10^4^ in case of the larvae reared in MCC. Incidentally, the percent distribution of *S. odorifera* in the midgut of these larvae was increased, probably due to the elimination of the most of the other gut flora after high temperature exposure. The observation of the natural habitat when ambient temperature was 39°C was analogous to the observations of heat shock treatment where the total bacterial population in the larval gut was reduced along with elevated relative presence of *S. odorifera* biogroup II.

Transstadial transmission and selective retention at high temperature qualified *S. odorifera* to be specially considered for the further studies. Understanding its interaction either with the midgut tissue of the adult *Ae. aegypti* or with the arbovirus, or both and its subsequent influence on the vector competence was the focus of further investigations.

### *Serratia odorifera* Enhances DENV-2 Susceptibility of *Ae. aegypti*

Studies were designed to understand the effect of *S. odorifera’s* presence in the midgut of *Ae. aegypti* on its susceptibility to DENV-2. For an individual experiment, *Ae. aegypti* females were split into three groups based on the type of blood meal they received. Group one received DENV-2 with no bacteria in the blood meal whereas, groups two and three were co-fed with *S. odorifera* and *M. oxydans* respectively, along with DENV-2 via blood meal. The data obtained in three independent experiments showed similar pattern.

On the 14^th^ post infection day (PID), the DENV-2 infection in the infected females was demonstrated by immunofluorescence assay and DENV-2 titer was determined by plaque assay. Immunofluorescence assay revealed that at 14 PID, the group receiving *S. odorifera* along with DENV-2 showed significant increase in the susceptibility to DENV-2 (39.4%±8.4; data of four independent experiments) as compared to the groups that received only dengue-2 (21.6%±1.2; data of three independent experiments) and *M. oxydans* (18.9%±4.4; data of three independent experiments). An ANOVA-one way analysis showed that the means were significantly different for the dissemination rates among the three groups (P = 0.0016). The dissemination rates between dengue-2 and dengue-2+ *S. odorifera* groups (Mann-Whitney U test P<0.05) as well as dengue-2+ *M. oxydans* and dengue-2+ *S. odorifera* groups (Mann-Whitney U test P<0.05) were significantly different. Whereas the dissemination rate did not vary significantly between dengue-2 and dengue-2+ *M. oxydans* groups (Mann-Whitney U test P>0.05).

It was important to analyze the DENV-2 titers in all the groups immidiately after blood meal and on 14^th^ PID. The titers were determined in individual mosquito carcasses. Immediately after blood meal, *Ae. aegypti* females had an average dengue-2 titer of 8.83×10^2^±1.92×10^2 ^PFU/mosquito for the first group and 8.61×10^2^±1.56×10^2 ^PFU/mosquito and 7.62 ×10^2^±2.77×10^2 ^PFU/mosquito for second and third groups respectively, indicating almost equal number of viruses were ingested by all the groups (Mann-Whitney U test P>0.5). At 14 PID, no significant difference in the virus titers was observed amongst three groups as the titer for the first group was 7.13×10^4^±9.97×10^3 ^PFU/mosquito whereas for second and third groups it retained at 6.86×10^4^±1.88×10^4 ^PFU/mosquito and 6.51×10^4^±1.83 ×10^4^ PFU/mosquito (Mann-Whitney U test P>0.5) respectively. Virus titers in dengue-2 negative mosquitoes were undetectable. These observations suggested that the presence of *S. odorifera* influenced the susceptibility of *Ae. aegypti* to DENV-2 specifically as more number of mosquitoes were affected with DENV-2 in *S. odorifera* fed group.

This led to the speculation that *S. odorifera* modulated *Ae. aegypti* susceptibility either through its direct interaction with virus or virus-specific gut receptor component or both. Therefore, it was essential to identify the repertoire of polypeptides of either bacteria or mosquito, interacting with the virus to understand the causal mechanism of enhanced vector susceptibility.

### Interaction of *S. odorifera* with the Gut Brush Border Membrane Fraction (BBMF) of *Ae. aegypti*

**Figure 1:**
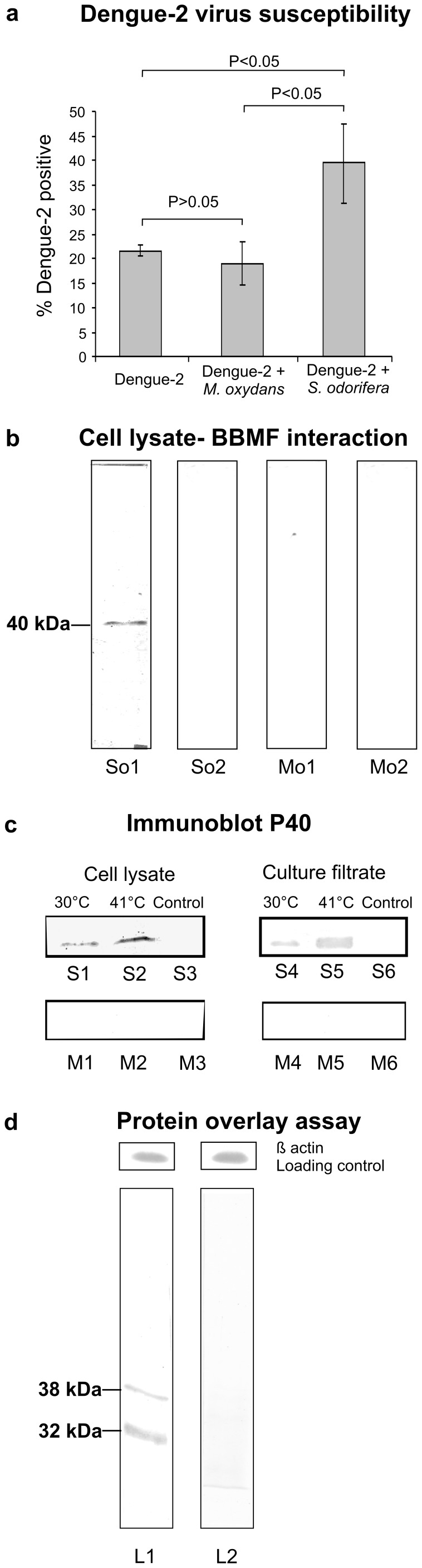
*Serratia odorifera* and *Ae. aegypti* interaction. a. Significance of *S. odorifera*’s presence in the blood meal on the DENV-2 susceptibility of *Ae. aegypti*: Adult females were fed with Blood + DENV-2, Blood + DENV-2+ *M. oxydans* (Blood + DENV-2+ *S. odorifera* via blood meal. DENV-2 dissemination was detected in the head squashes on 14 post infection days by IFA. The presence of *S. odorifera* in the blood meal significantly enhanced the dengue virus susceptibility (Mann-Whitney U test; P<0.05) compared to *M. oxydans* (Mann-Whitney U test; P>0.05). Post feeding virus titers in the blood meal were determined by plaque assay (1.8× 10^5^ PFU/mL of blood). b. Overlay assay with *S. odorifera* and *M. oxydans* cell lysates: The bacterial cell lysates of *S. odorifera* (So1 and So2) and *M. oxydans* (Mo1 and Mo2) were separated by SDS-PAGE and transferred to a nitrocellulose membrane. The membrane was overlaid with BBMF of *Ae. aegypti.* The putative binding proteins were detected by mouse anti-BBMF antibody and HRP labeled-secondary antibody. c. Expression of P40 in cell lysates and cell supernatants under different temperature conditions: *S. odorifera* cell lysate (S1 and S3 30°C, S2 41°C), *M. oxydans* cell lysate (M1 and M3 30°C, M2 41°C), culture filtrate of *S. odorifeara* (S4 and S6 30°C, S5 41°C) and culture filtrate of *M. oxydans* (M4 and M6 30°C, M5 41°C) were separated by SDS-PAGE and transferred to Hybond-C membranes. The membranes were incubated with the anti P40 mouse IgG (lanes S1, S2, S4, S5, M1, M2, M4 and M5) and with PBS pH 7.4 (lanes S3, S6, M3 and M6). Presence of P40 was detected by incubating the membranes with the secondary antibody (peroxidase-conjugated goat anti mouse IgG). Reaction was developed using H_2_O_2_ and DABT.d. Protein-protein interaction between BBMF and *S. odorifera* cell lysate: The membrane proteins of *Ae. aegypti* midgut (Lanes L1, L2 ) were separated by SDS–PAGE and transferred to Hybond-C membranes. The membranes were incubated with *S. odorifera* cell lysate (L1) and PBS pH 7.4 (L2) at 37°C. The putative P40 binding proteins were visible after incubation with anti P40 mouse antibody and the secondary antibody (peroxidase-conjugated goat anti mouse IgG). The reaction was developed using H_2_O_2_ and DABT. The molecular weights of DENV-2 binding proteins are shown on the left side of the blot.

Overlay assays were performed to investigate the interaction between *S. odorifera* and *Ae. aegypti* midgut BBMF. *S. odorifera* and *M. oxydans* cell lysates were transferred to hybond C membrane and overlaid with *Ae. aegypti* BBMF. Overlay assays revealed that 40 kDa polypeptide (P40) of *S. odorifera* interacted specifically with BBMF (Fig. 1b). However, none of the polypeptides of *M. oxydans* showed interaction with BBMF although it is a cohabitant with *S. odorifera* in most of the larval/adult midguts (Fig. 1b). P40 was also detected in the culture supernatant of *S. odorifera* (Fig. 1c). The expression of P40 in culture filtrate and cell lysate of *S. odorifera* was significantly enhanced after heat shock treatment (Fig. 1c). The peptide mass fingerprint of P40 by MALDI-TOF/TOF showed significant homology to the putative periplasmic protein of *Salmonella typhi* and exported protein of *Serratia marcescens*. Domain analysis of 65 amino acids showed the presence of N- myristoylation site, N-glycosylation site and casein kinase II phosphorylation site. Glycoprotein staining of *S. odorifera* cell extract revealed that P40 was a glycoprotein.

### Identification of P40 Interacting Polypeptides from *Ae. aegypti* Midgut BBMF

When SDS-PAGE separated polypeptides from *Ae. aegypti* BBMF were incubated with *S. odorifera* cell lysate and the interaction with P40 was detected by anti P40 antibody, two polypeptides of the midgut BBMF with molecular masses 32 kDa and 38 kDa were recognized as P40 binding proteins (Fig. 1d). The polypeptides interacting with P40 were further characterized using MALDI-TOF/TOF analysis. They were identified as prohibitin (**ABF18314**) and porin (**ABF18270**) respectively (For details see Table S2).

### Identification of P40 Interacting Regions on the Midgut Tissue

**Figure 2:**
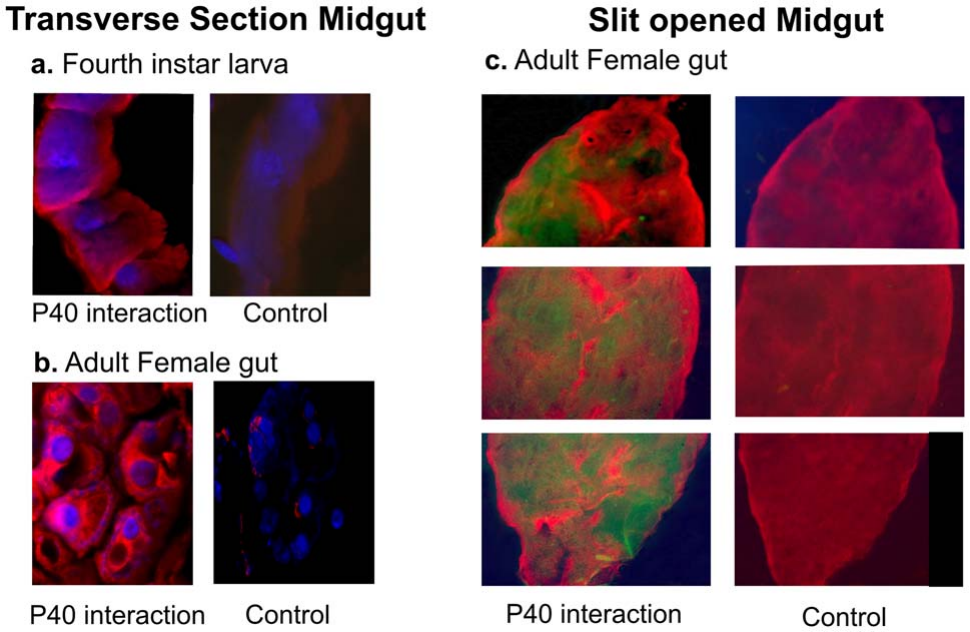
P40 localization in the *Ae. aegypti* gut. The midgut sections (10 µm) of *Ae. aegypt*i fourth instar larvae (a) adult female (b) and slit opened gut of adult females (c) were incubated with *S. odorifera* cell lysate and control midgut sections were incubated with PBS (pH 7.4). P40 interaction with the midgut epithelium was detected using mouse anti-P40 antibody and with a Cy3 conjugated rabbit anti mouse IgG secondary antibody. The signal was detected using a Zeiss microscope equipped with the Axiovesion detection system.

 Earlier experiments using BBMF revealed that P40 interacts with midgut extract of *Ae. aegypti*; it was therefore essential to demonstrate that these observations also hold true for dissected midgut tissues. Immunofluorescence microscopy confirmed the P40 interactions predominantly with columnar epithelial cells in the posterior region of the dissected midgut tissue (Fig. 2a, 2b and 2c).

### *Serratia odorifera* Polypeptides Interacting with DENV-2

**Figure 3:**
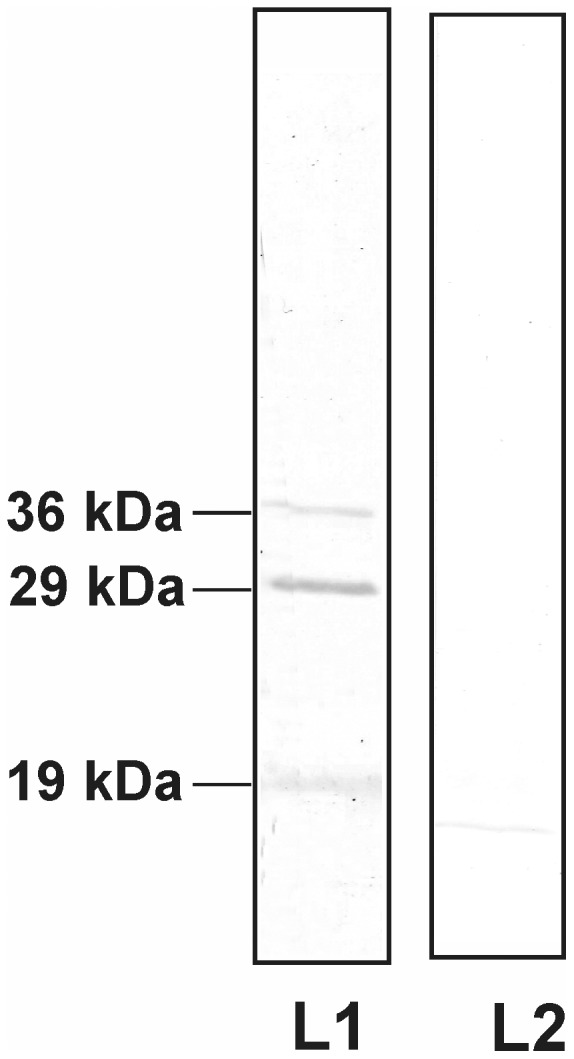
Virus overlay protein binding assay (VOPBA). *Serratia odorifera* cell lysate (lanes L1 and L2) were subjected to SDS-PAGE and transferred to Hybond-C membranes. Lane L1 was incubated with 5×10^5^ plaque-forming units of DENV-2, and lane L2 with PBS (pH 7.4) at 37°C. The putative DENV-2 binding proteins were visible after incubation with a rabbit polyclonal antibody to DENV-2 and a secondary antibody (peroxidase-conjugated goat anti-rabbit IgG). The reaction was developed using H_2_O_2_ and DABT. The molecular weights of dengue-2 binding proteins are shown on the left side of the blot.

 Virus overlay protein binding assays (VOPBA) were performed to investigate the repertoire of *S. odorifera* polypeptides interacting with DENV-2. When immobilized *S. odorifera* cell lysate was incubated with native DENV-2, three polypeptides of molecular masses 19 kDa, 29 kDa and 36 kDa of *S. odorifera* were recognized as DENV-2 binding proteins (Fig. 3a). However, P40 was not involved in this interaction. Putative identification of these proteins was carried out using MALDI-TOF/TOF analysis. A 36 kDa protein showed homology to DNA directed RNA polymerase subunit alpha of *Enterobacter* sp. (Strain 638) whereas 29 kDa polypeptide showed homology to Ribosomal RNA large subunit methyl transferase of *Xanthomonas oryzae* pv. Oryzae (For details see Table S3).

## Discussion

In the current study, we analyzed the bacterial diversity from the midgut of *Ae. aegypti* larvae obtained from a variety of habitats. The majority of these bacteria were Gram negative rods which were consistent with observations reported in earlier studies of field collected and laboratory reared mosquitoes [2], [3], [4], [5], [6], [7], [8], [9], [10], [11], [12], [13], [14], [15] suggesting that the midgut of mosquitoes have conducive conditions for these microbes, especially those belonging to the Gamma-proteobacteria family. This strong bias could be partly attributed to the LB/NB agar-based aerobic culturing method that was used for their cultivation. The LB/NB agar-based culturing method has some limitations in providing the complete composition of the mosquito midgut microbiota since a large fraction of bacteria are likely to be unculturable, similar to the human intestinal microbiota. High throughput sequencing-based metagenomic approaches are likely to provide comprehensive information on the total composition of the midgut microbiota.

Analysis of the microbial community in the larval habitat suggested that indeed, the larval surroundings influence the type of midgut microbes they harbor and only some of them are selectively retained as midgut inhabitants. Such information is not available in the studies conducted so far about the mosquito midgut inhabitants. In the current study, *S. odorifera* was found to be the most prevalent larval midgut inhabitant irrespective of the source of collection. Gusamo et al. [10] have also identified *Serratia* sp. in the diverticulum of *Ae. aegypti* mosquitoes. *Serratia marsescence* has been shown to be present in the midguts of field collected and laboratory reared *Anopheline* mosquitoes. [7], [10], [19]. Other *Serratia* sp. have also been isolated from the midguts of field collected and laboratory reared *Anopheles* adults [2], [5], [12]. Wang et al. [14] have also demonstrated an increase in the population of bacteria belonging to Enterobactereceae family consisting of genera *Serratia*, *Enterobacter* and *Klebsiella* after feeding blood meal to Anopheline females. The authors speculated that these bacteria are capable of coping with oxidative stress in the bolus [14]. These observations suggested that *Serratia* is commonly found in the midguts of mosquitoes.

It is believed that the gut undergoes sterilization process during the transition from larvae to adult and the emerged adults recruit new microbiota [19]. Interestingly, there are some reports which suggest otherwise that the gut does not undergo complete sterilization during transition, rather a part of the larval midgut is retained during the transformation to adult, and certain bacteria are also retained through metamorphosis [12]. Wang et al. [14] have extensively profiled the gut microbial consortium during different developmental stages of *Anopheles gambiae* and demonstrated that the profile changes drastically during the transition from pupae to adult but does not undergo complete sterilization, rather members of Enterobactericeae family predominate in the emerging adults. The observations of the current study corroborated with these later reports as *S. odorifera,* belonging to Enterobacteriaceae family was transstadially transmitted into the emerging imagoes whereas other gut microbes were eliminated during metamorphosis. Interestingly, *S. odorifera* also sustained in the midgut during exposure of larvae to higher temperature whereas other gut inhabitants were eliminated and the results correlated with the observations made in the natural habitat during summer season.

The correlation between higher ambient temperature and occurrence of viral epidemic has been reported earlier [20]. It was observed that the mosquitoes reared in insectaries at higher ambient temp have more virus load [21]. It has been also documented that climate variability and global warming are the important factors which may favour epidemics of dengue [22] probably due to the influence of these factors on the extrinsic incubation period. A study by Descloux et al. [23] demonstrates a correlation between epidemic dynamics of dengue in Nauomea and higher ambient temperature by analyzing the data of 40 years. Similarly, Chowell et al. [24] have also suggested significant impact of change in mean temperature on dengue epidemics in the jungle and coastal areas of Peru. Based on the observations of the current study, it can be speculated that elevated environmental temperatures can potentially favour the selective retention of *S. odorifera* in *Ae. aegypti* midgut and in turn can increase it’s susceptibility to DENV-2. The biochemical characterization of *S. odorifera* revealed that it has strong hemolytic and proteolytic properties which could be of special significance in the blood digestion in the midgut of adult *Ae. aegypti* females. It was speculated that *S. odorifera* with its unique qualities might play a critical role in vector competence of *Ae. aegypti* as it is the part of the same midgut milieu where vector and arbovirus interact.

The influence of *S. odorifera* on DENV-2 susceptibility was assessed by co-feeding DENV-2 with *S. odorifera* to aseptic *Ae. aegypti* mosquitoes. It resulted in the enhanced susceptibility of *Ae. aegypti* to DENV-2. Although, the virus titers among the three groups did not change significantly on 14^th^ PID as compared to initial titers, the number of mosquitoes getting dengue-2 infection increased significantly when *S. odorifera* was present in the blood-meal. A similar observation was reported by Mourya et al. [25] when *Aeromonas* sp. and *E. coli* fed *Ae. aegypti* showed enhanced susceptibility to DENV-2; underlying mechanism, however, was not identified. On the other hand, studies on the gut microbes of *Anopheline* mosquitoes have demonstrated the negative influence of these microbes in parasite transmission [5], [7], [13] mediated through the generation of reactive oxygen species and triggering of immune response due to the number of bacteria introduced in the gut. A recent report by Cirimotich et al. [13] correlated reactive oxygen species generating capabilities of *Enterobacter* species, a midgut inhabitant of field caught *Anopheles* mosquito, with its relative refractivity to Plasmodium transmission by field caught *Anopheles* mosquitoes. Joyce et al. [15] showed that in- vitro incubation of La Crosse virus with a mixed mosquito midgut bacterial population decreased the infectivity of LACV to Vero cells. Although in this study, the microbes were isolated from the midguts of *Ae. albopictus*, the effect was demonstrated on cultured Vero cells. It would be therefore essential to show the similar effect in live mosquitoes.

In the report by Pumpuni et al. [5] the negative effect on transmission of the parasite has been correlated with the presence of 3×10^8 ^CFU/ml of specific bacteria in the midgut of mosquitoes. However, it was subsequently concluded that the high bacterial counts in the blood meal evoked the host immune response which blocked the parasite development. There is a likely possibility that, the bacterial abundance in the midgut could create a physical barrier for parasite-receptor interaction. We used 3×10^4 ^CFU/mL bacteria in the feeding experiments, which matched numerically to the natural inhabitant bacteria in the mosquito midgut and hence did not encounter problems seen by other authors, on the contrary, observed the enhanced DENV-2 susceptibility.

It was speculated that the altered vector susceptibility by this gut inhabitant could be due to its interactions with either midgut tissue proteins or DENV-2 or both. Indeed, overlay assays confirmed the interaction of *S. odorifera* with BBMF through its 40 kDa polypeptide, P40 although; it failed to interact with DENV-2. Consequently, *Ae. aegypti* BBMF proteins prohibitin and porin were identified as P40 interacting proteins. In earlier reports VOPBA showed involvement of four proteins of BBMF of *Ae. aegypti* viz. actin, prohibitin, tubulin and vav-1 in DENV-2 interaction [26]. Interestingly, prohibitin was the unique target for both P40 and DENV-2. These results suggested that prohibitin modulated the susceptibility by interacting directly with DENV-2 and titrating out the viral load. Blocking of this molecule by P40 peptide of *S. odorifeara*, probably allowed more number of viruses to attach to their natural receptors leading to increased viral titers.

It has been suggested that microbes may have evolved strategies to manipulate host mitochondrial defense mechanisms, and any alterations in mitochondrial function may affect the immune response of cells to virus infection. Sharma and Quadri [27] have reported that Vi polysaccharide of *Salmonella typhi* targets the prohibitin family of molecules (Prohibitin-1 and Prohibitin-2) in the intestinal epithelial cells and suppresses the early inflammatory responses. *S. odorifera* P40, a glycoprotein, might be playing similar role like Vi polysaccharide by repressing early immune response of *Ae. aegypti*.

Prohibitin is the highly conserved protein in eukaryotes having a multifaceted role in cell physiology [26], [28]–[32]. Changes in the abundance and localization of mitochondrial proteins such as Toms, VDACs and prohibitins are linked to the number of factors related to immune response, antiviral state, and viral infection [33]. It has been documented that prohibitin expression increases after infection with Human Respiratory Syncytial Virus [34], H9N2 [35], hepatitis B virus [36] and hepatitis C [37]. It activates complement pathway [32] and shares a number of structural features/functional domains with Rel-1 and other immune related molecules (see Table S4) although its role in insect immunity is yet unknown. Functional site analysis of the *Ae. aegypti* prohibitin molecule revealed that it has similar functional sites to Rel-1 which makes it a potential immune response molecule like Rel-1. It is reported that the activation of the toll immune signaling pathway by dengue infection is strongly supported by the up-regulation of Rel1 and its downstream antimicrobial peptides [38].

Recently, Kuadkitkan et al. [39] showed that DENV-2 interacts with prohibitin molecule and mediates its entry in insect cells. The observations of the current study also showed interaction between DENV-2 and prohibitin. However, our study indirectly demonstrated that prohibitin is a refractivity conferring non-receptor molecule rather than receptor as proposed by Kuadkitkan et al. [39]. These authors showed the role of this molecule as the receptor for dengue-2 by demonstrating antibody mediated inhibition of infection and siRNA mediated knockdown of prohibitin expression which led to significant reduction in the virus production in both *Ae. aegypti* and *Ae. albopictus* cell lines. Earlier reports have shown that prohibitin has immune related function [32], [34]–[37]. It is also suggested that the depletion of prohibitins can cause impairment in diverse cellular functions and may lead to the increased levels of reactive oxygen species (ROS) [40] which are known to be responsible for mitochondrial antimicrobial defense system [33]. Cirimotich et al. [13] demonstrated that the generation of reactive oxygen species by gut bacteria of *Anopheline* mosquito has significant anti-Plasmodium effect. The observed inhibition of DENV-2 in prohibitin knock down *Aedes* cells by Kuadkitkan et al. [39] could be potentially due to either elevation of ROS or impairment in normal cellular function as a consequence of the significant reduction in prohibitin levels. It would be therefore essential to demonstrate this impact of prohibitin knock down in adult Aedes females.

 Our report demonstrates the role of a gut microbe of *Ae. aegypti* in enhancing its arbovirus susceptibility along with the underlying mechanism. This study also identifies the host target proteins and their respective roles in modulating DENV-2 susceptibility. Prohibitin has been shown to be the versatile molecule and an important player in the immune response mechanism of higher eukaryotes. The observations of this study demonstrate its unique role in viral susceptibility of the mosquito hosts. Further investigations would be essential to decipher its multifaceted role in this insect host.

## Materials and Methods

### Collection of Mosquitoes and Isolation of Bacterial Flora from their Midguts

The third and fourth instar larvae of *Ae. aegypti* used in this study were obtained from three laboratory reared colonies and two natural habitats. The laboratories maintaining mosquito colonies were: 1. Microbial Containment Complex (MCC), NIV, Pune; 2. National Chemical Laboratories (NCL), Pune; 3. Entomology group at the Armed Force Medical College (AFMC), Pune, whereas, the household containers in the city of Pune and Ahemedabad were natural habitats. The fourth instar larvae collected from the fields were brought to the laboratory and their identity was confirmed by morphological analysis to be ***Ae. aegypti*** **and** were then used in the study. The larvae were surface sterilized for five seconds in 70% ethanol. The larval guts were dissected aseptically in laminar hood. The dissected midguts were pooled and transferred to 100 **µ**l of sterile phosphate-buffered solution (PBS pH 7.4) and were homogenized. The homogenates of the midguts were serially diluted and were plated on the nutrient agar plates. All the plates were incubated at 30°C for 24 h. The midguts of the pupae and imagoes were also processed similarly for the isolation of gut microbes. Each pool of midguts had five guts of either larvae, pupae or imagoes.

### Identification of the Midgut Microflora

A single colony isolation technique was used for all the morphologically distinct colonies visible on the nutrient agar plate. The bacterial identification was done by the conventional morphological and biochemical characterization, following the protocol in Bergy’s manual of determinative bacteriology [41]. These identifications were further confirmed by 16S rDNA sequence comparisons [42].

####  DNA sequencing

Genomic DNAs were isolated from the different bacterial isolates by the phenol extraction method and a stretch of 1100 bp 16s rDNA gene region was PCR amplified using the universal 16s rDNA gene specific primers. The purified PCR products were sequenced using ABI prism 3730 sequencer (Applied Biosystems, USA) and Big dye terminator sequencing kit (ABI Prism, USA). The sequences were analyzed by BLAST tool [43]. These sequences have been deposited in Genbank (Table1).

### High Temperature Exposure of the Larvae

The higher ambient temperature during summer season, raises the temperature of the water body which is the natural habitat of the mosquito larvae. To understand the status of larval midgut inhabitants during this season, larval collection was made from water bodies in the city of Pune when the temperatures reached the range of 39–42°C. To simulate the similar effect in the laboratory, the mosquito larvae were exposed to high temperature (42°C) as described by Patil *et al.,* [44]. At the end of exposure time, the larvae were dissected to isolate the midguts and the isolation and identification of the gut flora was carried out as mentioned in the earlier sections.

### Transstadial Transmission

The aim of this exercise was to understand the status of gut microflora during larval metamorphosis into an adult. For this study, fourth instar larvae and pupae from other laboratories and fields were brought to our laboratory and were maintained under standard laboratory conditions in clean water surroundings and with a change of water every 24 hours till the larvae metamorphosed into adults. The developing pupae were transferred to new containers and emerging imagoes were not offered any food. Their midguts were dissected aseptically within 24 hours of emergence for the isolation and identification of bacterial flora and prior to dissection; the imagoes were surface sterilized with 70% alcohol to avoid any microbial carry over from their surroundings.

### Virus Stock Preparation

Dengue-2, Trinidad (TR1751) virus stock was prepared in infant Swiss albino mice (1–2 days old) as per the protocol described by Ilkal *et al.,* [45]. In order to prepare the virus stock, DENV-2 suspension was initially reconstituted in 0.5 mL distilled water, and the dilutions were prepared in 1.25% bovine serum albumin phosphate saline (BAPS), pH 7.4. One to two day old mice were inoculated intra-cerebrally with 0.02 mL of the DENV-2 suspension (8×10^7^ PFU/mL). The mice were monitored for sickness. After about 8–10 days, when the mice showed dengue-2 sickness, they were sacrificed, and their brains were homogenized using 1.25% BAPS (20% w/v). The homogenates were centrifuged at 16,000 g for 60 minutes. The supernatants were distributed in glass vials, lyophilized and stored at −20°C. The virus titer (5×10^7^ PFU/mL) of one of the vials was determined by plaque assay.

### Isolation of the Gut Brush Border Membrane Fraction (BBMF) from *Ae. aegypti*

 The BBMFs from the midgut epithelial cells of *Ae. aegypti* larvae and adults were prepared as described by Mourya *et al.,* [46]. The adult females were chilled on ice, the midguts were extruded by dissection under a binocular microscope, peritrophic membranes and malpighian tubules were removed, and the midguts were rinsed in ice-cold buffer A (0.3 M mannitol/5 mM EGTA, 20 mM Tris/HCl, 2 mM PMSF, pH 7.4). The midguts were then placed in 1.5-mL cryogenic vials and stored at −80°C until required. For the preparation of the BBMF, the midguts were homogenized in buffer B (0.3 M mannitol, 5 mM EGTA, 20 mM Tris/HCl, 2 mM PMSF, Triton X-100, pH 7.4). The samples were allowed to stand on ice for 20 minutes and then centrifuged at 2,000 g for 15 minutes at 4°C. The supernatants were collected and kept on ice. The pellets were re-extracted in buffer B. The supernatants from both the extractions were centrifuged at 23,200 g for 60 minutes at 4°C. The pellets were resuspended in 1× PBS with PMSF (2 mM) such that the final dilution is 1 midgut/µl.

### P40 Antigen Preparation

After electrophoretic separation of *S. odorifera* cell lysate by SDS-PAGE, the gel piece corresponding to 40 kDa polypeptide was excised from the gel and washed with normal saline. The gel piece was then homogenized in 1 mL of normal saline for immunization, centrifuged and purity of the 40 kDa protein was reconfirmed by SDS-PAGE before injecting it into mice (Fig. S1).

### Immune Sera Preparation Against DENV-2/BBMF/P40 in Mice

Three-four weeks old mice were inoculated intra-peritonealy with dengue-2/BBMF/P40 and were maintained under standard laboratory conditions. Booster doses of the antigen along with Freund’s incomplete adjuvant (1∶1) were given (one dose/week) for two weeks. The mice with higher titers of the antibodies were injected with 10% ascitic tumor cells, intra-peritonealy. The intra-peritoneal fluid was collected on the 5^th^ PID and after removal of the debris by centrifugation, the supernatant was tested for the presence of antibodies. Anti DENV-2 and anti P40 serum were adsorbed with the mosquito midgut extract. Anti BBMF serum was adsorbed with *Serratia* cell lysate. The antibodies were then purified using protein A column. The pre-immune serum was collected one day prior to the immunizations.

### Introduction of the Midgut Bacterial Isolates Along with DENV-2 in the Blood Meal of *Ae. aegypti* Females

#### Antibiotic treatment

The resident gut flora of the *Ae. aegypti* mosquitoes were cleared using the tetracycline treatment as all the cultivable bacteria isolated were found to be sensitive to tetracycline. First instar larvae were maintained in water having 0.25 mg/mL tetracycline till the emergence of the adults. The cages were wiped with 70% alcohol before placing the pupae. The emerging adult mosquitoes were fed with tetracycline (0.25 mg/mL) in sterile 10% sucrose solution on sterile cotton balls for three consecutive days and were then starved for 24 hours before receiving the blood meal [47].

#### Oral feeding of mosquitoes

The virus infection assays were performed with 4–6 days old *Ae. aegypti* females. The white leg horn fowls were bled through the heart, and the blood was defibrinated using glass beads and was used for further feeding experiments. The mosquitoes were allowed to feed for one hour through a goat intestine membrane covering the base of a glass feeder that carried the blood-virus or blood-virus-bacteria mixture maintained at 37°C. For an individual experiment, the infectious meal was given to three groups; Group 1: One mL of blood mixture containing the 250 µl dengue virus (5×10^5^ PFU/mL) [Total n = 78, about 25–30 females/experiment in three independent experiments], Group 2: One ml of blood mixture containing the 250 µl dengue virus (5×10^5^ PFU/mL) along with 250 µl of *Serratia odorifera* (1.2×10^5^ CFU/mL) [Total n = 158, about 35–40 females/experiment in four independent experiments] and Group 3: One mL of blood mixture containing the 250 µl dengue virus (5 ×10^5^ PFU/mL) along with 250 µl of *Microbacterium oxydans* (1.2×10^5^ CFU/mL) [Total n = 87, about 25–30 females/experiment in three independent experiments]. Fully engorged females were transferred to small containers and were maintained with 10% glucose at 28±1°C for 14 days. To evaluate the infection and dissemination rate and in turn vector competence on 14 PID, surviving females were sacrificed by transferring them to −80°C and tested for the presence of dengue-2 antigens in the head squashes by immunofluorescence assay (IFA). Three such independent experiments were carried out for evaluating the data. The DENV-2 titer in mosquitoes at 0 hour was determined by plaque assay (n = 10 for each group). Similarly, the dengue-2 titer in individual carcass of infected female was determined using a plaque assay. The average of virus titers from 24 carcasses of each group was determined and used for comparative analysis.

### Detection of the Dengue Viral Antigen in the Head Squashes by the Indirect Immunofluroscence Assay (IFA)

 The presence of the viral antigen was determined by the IFA test on the14^th^ post infection day (PID) by modifying the procedure described by Mourya and Mishra, [48]. The head squashes were prepared on glass slides. The slides were immersed in blocking buffer (0.1% Tween 20 and 2% BSA in PBS) for 1 hour at room temperature; the slides were then incubated with anti DENV-2 mouse antibodies, followed by FITC-conjugated goat anti-mouse IgG for 1 hour each at 37°C. These slides were mounted with ProLong Gold anti-fade reagent with Evans Blue, and were visualized under a fluorescent microscope. For each experiment, positive and negative controls were processed using the same protocol. These experiments were repeated three times and the statistical significance was determined by calculating ANOVA with Tukey’s HSD and further confirmed the statistical significance by analyzing the data with non-parametric test Mann Whitney U test.

#### Plaque assay

The DENV-2 copy number in the carcasses of dengue positive mosquitoes was determined using plaque assay. An individual mosquito was titurated in 1 mL of Mitsuhashi and Maramorosch medium to release infectious virus. C6/36 cells were grown to confluent monolayers in 12-well plates, were infected with 10-fold serial dilutions of mosquito homogenates for 1 hour, and then overlaid with carboxymethyl cellulose-nutrient mixture. After five days incubation at 37°C, the cells were stained with crystal violet solution. The viral titers were determined by counting plaques. The virus titer in the individual mosquito is reported as plaque forming units (PFU) per mosquito (values are expressed as the means±SD). The virus titers in the post blood meal fed mosquitoes was determined using the same protocol.

### Bacterial Cultures and Cell Lysate Preparation

Pure cultures of *Serratia odorifera* and *Microbacterium oxydans* were grown in the nutrient broth at 37°C with constant shaking for 6 hours. The cells were centrifuged and resuspended in phosphate buffer (pH 7) and were lysed by sonication. The whole cell lysates were used for overlay assays.

### Overlay Protein Binding Assays

To understand the protein-protein interactions among virus or BBMF or *S. odorifera* cell lysates, overlay assays were performed. The protocol mentioned in this section is common for all the overlay assays. The brush border membrane proteins of *Ae. aegypti* or *S. odorifera* cell lysate (50 µg) were separated on SDS-polyacrylamide gel (SDS-PAGE) and were transferred to the nitrocellulose membrane Hybond C using a semi-dry blotting apparatus (Biorad Laboratories, USA) in 48 mmol Tris, 39 mmol glycine, and 20% (vol/vol) methanol. The membranes were blocked with 2% BSA (SIGMA) in PBST (phosphate buffered saline pH 7.4, 0.5% Tween 20) at 4°C overnight, and were washed three times 30 minutes each with PBST following incubation. Based on the type of study, the membranes were either incubated with the purified DENV-2 or BBMF or *S. odorifera* cell lysate in PBS at 37°C for 1 hour and were washed three times for 30 minutes each with PBST. The membranes were then incubated for one hour with respective antibodies (anti DENV-2 or anti BBMF or anti P40). After washing the membranes three times for 30 minutes each with PBST, the membranes were incubated for 1 hour at room temperature in secondary antibody conjugated with peroxidase/alkaline phosphatase (diluted 1∶3000 in PBS). Finally, the membranes were washed three times 30 minutes each with PBST and were developed with H_2_O_2_ and DABT or NBT-BCIP solution depending on the secondary antibody conjugate.

### Protein Identification Using MALDI-TOF/TOF MS

The spots corresponding to the protein of interest were excised from SDS-PAGE gels and subjected to alkylation followed by in-gel digestion with trypsin. The masses of resultant peptides were analyzed by matrix-assisted laser desorption/ionization time of flight (MALDI-TOF/TOF) on Ultraflex TOF/TOF (Bruker Daltonics, Germany). MALDI TOF/TOF analysis was carried out in Indian Institute of Science, Bangalore. The mass spectrum generated by each sample was searched against protein databases (NCBInr, MSDB, and Swissprot) using the MASCOT search engine, and the proteins were identified based on homology.

### In-vitro Interaction of *S. odorifera* P40 with Midgut Epithelium of *Ae. aegypti*

The midgut epithelium of *Ae. aegypti* adult female mosquito was exposed by dissecting the gut longitudinally, and the tissue was fixed in 4% formaldehyde. The fixative was removed by thorough washing and was incubated with *S. odorifera* cell extract for 30 minutes. After washing, the tissues were covered with mouse anti P40 antibody (1∶200) followed by incubation with cy3-conjugated rabbit anti-mouse (1∶500) antibody. The nuclei were stained with DAPI followed by washing. The midgut tissues were then mounted in 50% glycerol and visualized by fluorescence microscope (Zeiss Axioskop equipped with camera and AxioVision software). Same exposure time was used for the control and the experimental slides while capturing the images. The negative control was also processed using the same protocol as above, where the gut tissues were overlaid with PBS pH 7.4.

### Statistical Analysis

Analysis of variance (ANOVA) was used to evaluate the effect of presence of different midgut bacteria on DENV-2 dissemination rate. The groups were also compared by nonparametric Mann-Whitney U test for confirmation of the results. The viral loads were log-transformed for improvement of the normality and compared by non-parametric Mann-Whitney U test with the significance levels set at p<0.05.

## Supporting Information

Figure S1 **Western blotting analysis for P40.** Cell lysate of *S. odorifera* was subjected to two dimensional gel electrophoresis and transferred to nitrocellulose membranes. P40 was visualized after incubation with a mouse polyclonal antibody to P40, and a secondary antibody (peroxidase-conjugated goat anti-mouse IgG). Reaction was developed by H_2_O_2_ and DABT.
(TIF)

Table S1 **Mosquito midgut microflora studies.**
(DOC)

Table S2 **P40 binding proteins from brush border membrane of** ***Ae. aegypti.***
(DOC)

Table S3 **Dengue-2 virus binding proteins from** ***S. odorifera*** **cell lysate.**
(DOC)

Table S4 **Functional site comparison of prohibitin with other immunity proteins of** ***Aedes aegypti.***
(DOC)
