# Supplementary material for: Serratia odorifera a Midgut Inhabitant of Aedes aegypti Mosquito Enhances Its Susceptibility to Dengue-2 Virus
Source: PLoS One. 2012 Jul 27;7(7):e40401. doi: 10.1371/journal.pone.0040401 (PMC3407224; doi:10.1371/journal.pone.0040401)
Supplement: Table S1 — Mosquito midgut microflora studies. (DOC) [file pone.0040401.s002.doc]

**Table S1: Mosquito midgut microflora studies**

|  | **Mosquito** | **Bacteria** | **Remark** | **Reference** |
| --- | --- | --- | --- | --- |
| 1 | *An. arabiensis*  Field-collected mosquitoes | **Culture dependent**  *Bacillus simplex,Vibrio metschnikovii*  *Serratia odorifera* AJ233432, *Nocardia corynebacterioides, Bacillus silvestris* AJ006086, *Escherichia senegalensis, Janibacter limosus*  **Culture independent**  *Acidovorax temperans, Mycoplasma wenyonii, Stenotrophomonas maltophilia, Paenibacillus* sp. strain AY382189, *Anaplasma ovis* AF414870, *Ehrlichia* sp. strain Bom Pastor AF318023 | investigated for their suitability for a paratransgenic *Anopheles* mosquito | Lindh *et al*. 2005 |
| 2 | *An*. *gambiae* sensu stricto | **Culture dependent**  *Pseudomonas putida*  **Culture independent**  *Stenotrophomonas maltophilia, Stenotrophomonas* sp. strain AJ002814, *Aeromonas hydrophila* X87271, *Aeromonas* sp. strain U88656, *Aeromonas* sp. strain AF099027 | investigated for their suitability for a paratransgenic *Anopheles* mosquito | Lindh *et al*. 2005 |
| 3 | *An. funestus* | **Culture independent**  *Spiroplasma* sp. strain AB048263, *Spiroplasma* sp. strain AJ245996 | investigated for their suitability for a paratransgenic *Anopheles* mosquito | Lindh *et al*. 2005 |
| 4 | Malaria mosquito | *Pseudomonas* sp. |  | Jadin *et al.* 1966 |
| 5 | *An. stephensi* | *Asaia spp., Gluconobacter asaii, Acetobacter aceti*  *Sphingomonas rhizogenes* | Asaia sp. was abundant in the mosquito body, with bacterial counts of up to 9.8 × 10^5^ colony forming units (CFU) per female and 9.8 × 10^4^ CFU per male individuals. | Favia *et al.* 2007 |
| 6 | *An. maculipennis* | *Serratia spp., Asaia spp., Staphylococcus spp.* |  | Favia *et al.* 2007 |
| 7 | *An. gambiae* | *Sphingomonas spp., Phenilobacterium spp.*  *Asaia spp., Burkolderia spp., Aquabacterium sp.*  *Acinetobacter spp., Pseudomonas spp.* |  | Favia *et al.* 2007 |
| 8 | *Aedes stricticus* and *Aedes vexans* | *Spiroplasma sabaudiense* |  | Abalain-Colloc *et al*. 1987 |
| 9 | *Culex tritaeniorhynchus* | *Spiroplasma taiwanense* sp |  | Abalain-Colloc *et al.* 1988 |
| 10 | *Anopheles gambiae* and *An. funestus* | *Escherichia coli, Enterobacter* agglomerans  total 73 bacterial isolates | *An. funestus* females that harbored gram positive bacteria were more likely to be infected with sporozoites compared with those with no cultivable bacteria or gram negative bacteria in their midguts. | Straif *et al*. 1998 |
| 11 | *Anopheles albimanus* and  *A. stephensi* | *Serratia marcescens,, Enterobacter cloacae* ,*Enterobacter amnigenus* 2, *Enterobacter* sp., and *Serratia* sp. was recovered in field samples of 1998  *Pseudomonas aeruginosa*  *Enterobacter* sp. was recovered in field samples of 1997 |  | Gonzalez-Ceron *et al*. 2003 |
| 12 | Larvae *Aedes aegypti* | *Spiroplasma taiwanense* |  | Humphery-Smith *et al.* 1991 |
| 13 | *Anopheles stephensi* | *Spiroplasma taiwanense* |  | Humphery-Smith *et al*. 1991 |
| 14 | *Aedes sollicitans*. | *Spiroplasma culicicola* sp. nov. |  | Hung *et al*. 1987 |
| 15 | *Culex quinquefasciatus* | **Culture dependent**  *Acinetobacter junii, Staphylococcus epidermidis, Stenotrophomonas maltophila, Microbacterium oxydans, Pantoea agglomerans, Acinetobacter calcoaceticus, Bacillus thuringiensis, Pseudomonas aeruginosa, Aeromonas culicicola*  **Culture independent**  *Enterococcus seriolicida, Lactococcus garvieae,*  *Lactococcus garvieae* strain FLG12, *Acinetobacter* sp. Aerobic And few un-culturable bacteria |  | Padiyar *et al*. 2004 |
| 16 | *Anopheles stephensi*. | *Escherichia coli* H243, *E. coli* HB101. *Pseudomonas aeruginosa*, and *Ewingella americana*) and two gram-positive (*Staphylococcus aureus* and *Staphylococcus epidermidis*) | All gram-negative bacteria strains partially or completely inhibited oocyst formation at different concentrations. | Pumpini *et al.* 1996 |
| 17 | Culex annulus | Spiroplasma diminutum sp |  | **Williamson *et al*. 1996** |
| 18 | *Anopheles stephensi, An. gambiae,* and *An. albimanus.* | *Pseudomonas cepacia, Enterobacter agglomerans,* and *Flavobacterium spp*. were found in all three anopheline species.  *Escherichia coli* HS5 |  | Pumpini *et al.* 1996 |
| 19 | *Anopheles gambiae* | *Enterobacter asburiae* , *Microbacterium* sp., *Sphingomonas* sp. E-(s)-e-D-4(2) , *Serratia* sp. , *Chryseobacterium meningosepticum* ,  *Asaia bogorensis* , *Bacillus subtilis*, *Enterobacter aerogenes*, *Escherichia coli*, *Herbaspirillum* sp., *Pantoea agglomerans*, *Pseudomonas fluorescens*, *Pseudomonas straminea*, *Phytobacter diazotrophicus* and *Serratia marcescens* | The mosquitoes' natural microbiota can influence their permissiveness to *Plasmodium* infection | Dong *et al*. 2009 |
| 20 | *Anopheles stephensi* lab reared | **Adult Male Culturable**  Chryseobacteriummeninqosepticum, Agrobacterium sp., Pseudomonas mendocina, Serratia marcescens  **Adult Male Unulturable**  C. meninqosepticum, Elizabethkingia meninqosepticum, A. Tumefaciens, P. tolaasii  Klebsiella sp., S. marcescens  **Adult Female Culturable**  C. meninqosepticum, Comamonas sp., P. mendocina  S. marcescens  **Adult Female Unulturable**  C. meninqosepticum, E. meninqosepticum  S. marcescens , Serratia sp. | bacterial diversity study | Rani *et al*. 2009 |
| 21 | **Field- collected** A. stephensi**.** | **Adult Male Culturable**  Micrococcus sp., Staphylococcus hominis, S. saprophyticus, Acinetobacter A. lwofii, A. radioresistens, A. johnsonii, Enterobacter cloacae  Escherichia hermani  **Adult Male Unculturable**  Bacillus sp., Paenibacillus alginolyticus, P. chondroitinus, Paenibacillaceae, Herbaspirillum sp.  Photorhabdus luminescens  **Adult Female Culturable**  Chryseobacterium indologenes,  Acinetobacter hemolyticus, A. radioresistens  Citrobacter freundii, Enterobacter cloacae,  E. sakazaki, E. hermani  **Adult Female Unculturable**  Leuconostoc citreum, Achromobacter xylosoxidans  Acinetobacter hemolyticus, Acinetobacter sp., Pseudomonas putida, P. synxantha  Pseudomonas sp., S. marcescens, S. nematodiphila  S. proteamaculans, Xenorhabdus nematodiphila  Leminorella grimontii  **Larvae Culturable**  C. indologenes, Bacillus sp., B. cereus  B. firmus, Exiguo bacterium, Acinetobacter venetianus, Aeromonas sobria, A. popoffii  P. anquilliseptica, Pseudoxanthomonas  Thorsellia anopheles, Vibrio chlorae, Deinococcus xinjiangensis  **Larvae Unculturable**  Calothrix sp., Brevibacterium paucivorans, Dysqonomonas sp., Staphylococcus cohnii, S. suis  B. thermo amylovorans, Lactobacillus  Azoarcus sp., Leptothrix sp., Hydroxenophaga  Ignatzschineria larvae sp., Enterobactersp., Serratia sp., Serratia sp., T. anopheles, S. Marcescens, S. Nematodiphila, D. xinjiangensis | bacterial diversity study | Rani et al 2009 |
| 22 | ***Anopheles stephensi, Anopheles gambiae, Aedes aegypti, and Aedes albopictus,*** | Asaia spp., Gluconacetobacter liquefaciens, Burkholderia sp. |  | Chouaia et al. 2010 |
| 23 | *Anopheles gambiae* | *Acinetobacter sp.* 8A12N2*, B. pumilus, Bacillus sp.* MW3*, Enterobacter sp., P. putida* strain NBAII CK-24 E*, B. cereus* strain NMRL PED1*, Bacillus sp.* “Mali 51”, *E. mexicanum* strain 6L6, *K. turfanensis* strain GJM817, *Pantoea sp.* CWB600, *P. rhodesiae* strain NO5, *Staphylococcus sp.* TP-Snow-C19, *Arthrobacter sp.* CY2W2, *Comamonas sp.* RV_A09_23b, *Enterobacter sp.* 1360, *Knoellia sp.* RCML-25 | anti-Plasmodium effect is largely caused by bacterial generation of reactive oxygen species. | Cirimotich et al. 2011 |
| 24 | **Field Collected** *Anopheles gambiae* | *Klebsiella, Raoultella, Serratia, Enterobacter, Aeromonas, Pseudomonas, Elizabethkingia, Acinetobacter, Comamonas, Propionibacterium, Stenotrophomonas, Bacillariophyta, Thorsellia, Finegoldia, Chlorophyta, Methylocystis, GpIIa, Roseomonas, Novosphingobium, Aerococcus, Corynebacterium, Lactobacillus, Cloacibacterium, Rhizobium, Porphyrobacter, Agromyces, GpV, Clostridium, Hydrogenophaga GpI, Methylophilus, Fusobacterium, Chryseobacterium, Pelagibacter, Sphingobium* | Gut bacterial composition at family level in different life stages of *An. gambiae* | Wang et al. 2011 |
| 25 | **Field Collected *Aedes albopictus*** | *Erwinia quercina, Vagococcus salmoninarium, Kluyvera cryocrescens, Enterobacter ludwigii , Pseudomonas rhodesiae , Pantoea agglomerans, Bacillus megaterium, Chryseobacterium aquaticum, Erwinia quercina, Roseomonas cervicalis, Pedobacter agri, Curtobacterium flaccumfaciens, Leuconostoc mesenteroides, Curtobacterium flaccumfaciens, Paenibacillus borealis, Brenneria quercina, Leuconostoc mesenteroides, Vagococcus salmoninarium, Brenneria salicis, Erwinia persicinus* | showed a significant reduction in infectivity of LACV for Vero cells. | Joyce et al. 2011 |

**REFERENCES**

Lindh, J.M., Terenius, O., Faye, I. (2005) 16S rRNA Gene-Based Identification of Midgut Bacteria from Field-Caught *Anopheles gambiae sensu lato* and *A. funestus* mosquitoes reveals new species related to known insect symbionts. *Appl Environ Microbiol*  **7**1:7217-7223.

Jadin J, Vincke IH, Dunjic A, Delville JP, Wery M, Bafort J, Scheepers-Biva M (1966) Role of *Pseudomonas* in the sporogenesis of the hematozoon of malaria in the mosquito. Bull Soc Pathol Exot Filiales 59:514-525.

Favia G, Ricci I, Damiani C, Raddadi N, Crotti E, et al. (2007) Bacteria of the genus *Asaia* stably associate with *Anopheles stephensi*, an Asian malarial mosquito vector. Proc Natl Acad Sci USA 104:9047-9051.

Abalain-Colloc, ML, Chastel C, Tully JG, Bove JM, Whitcomb RF, Gilot B, Williamson DL (1987) *Spiroplasma sabaudiense* sp. nov. from mosquitos collected in France. Int J Syst Bacteriol 37:260-265. 2

Abalain-Colloc ML, Rosen L, Tully JG, Bove JM, Chastel C, Williamson DL (1988) *Spiroplasma taiwanense* sp. nov. from *Culex tritaeniorhynchus* mosquitos collected in Taiwan. Int J Syst Bacteriol 38:103-107.

Straif SC, Mbogo CN, Toure AM, Walker ED, Kaufman M, Toure YT, Beier JC (1998) Midgut bacteria in *Anopheles gambiae* and *An. funestus* (Diptera: Culicidae) from Kenya and Mali. J Med Entomol 35:222-226.

Gonzalez-Ceron L, Santillan F, Rodriguez MH, Mendez D, Hernandez-Avila JE (2003) Bacteria in midguts of field-collected *Anopheles albimanus* block *Plasmodium vivax* sporogonic development. J Med Entomol 40: 371-374.

Humphery-Smith I, Grulet O, Chastel C (1991) Pathogenicity of *Spiroplasma taiwanense* for larval *Aedes aegypti* mosquitoes. Med Vet Entomol 5:229-232

Humphery-Smith I, Grulet O, Le Goff F, Chastel C (1991) *Spiroplasma* (Mollicutes: Spiroplasmataceae) pathogenic for *Aedes aegypti* and *Anopheles stephensi* (Diptera: Culicidae). J Med Entomol 28:219-222.

Hung SHY, Chen TA, Whitcomb RF, Tully JG, Chen YX (1987) *Spiroplasma culicicola* sp. nov. from the salt marsh mosquito *Aedes sollicitans*. Int J Syst Bacteriol 37:365-370.

Pidiyar VJ, Jangid K, Patole MS, Shouche YS (2004) Studies on cultured and uncultured microbiota of wild *Culex quinquefasciatus* mosquito midgut based on 16S ribosomal RNA gene analysis. Am J Trop Med Hyg 70:597-603.

Pumpuni CB, DeMaio J, Kent M, Davis JR, Beier JC (1996) Bacterial population dynamics in three *anopheline* species: the impact on *Plasmodium* sporogonic development. Am J Trop Med Hyg 54: 214-218.

**Williamson DL, Tully JG, Rosen L, Rose DL, Whitcomb RF, AbalainColloc ML, Carle P, Bove JM, Smyth H** (1996) Spiroplasma diminutum sp. nov., from Culex annulus mosquitoes collected in Taiwan. Int J Syst Bacteriol 46**:**229-233.

Dong Y, Manfredini F, Dimopoulos G (2009) Implication of the Mosquito Midgut Microbiota in the Defense against Malaria Parasites. PLoS Pathog 5: e1000423. doi:10.1371/journal.ppat.1000423.

**Rani A,  Sharma A,  Rajagopal R, Adak T,**  **Bhatnagar RK (2009)** Bacterial diversity analysis of larvae and adult midgut microflora using culture-dependent and culture-independent methods in lab-reared and field-collected Anopheles stephensi-an Asian malarial vector. BMC Microbiol  9**:**96doi:10.1186/1471-2180-9-96.

Chouaia B, Rossi P, Montagna M, Ricci I, Crotti E, damián C, Epis S, Faye I, Sagnon NF, Alma A, Favia G, Daffonchio D, Bandi C (2010) Molecular Evidence for Multiple Infections as Revealed by Typing of Asaia Bacterial Symbionts of Four Mosquito Species Appl. Environ. Microbiol. 76 (22) 7444-7450

Wang Y, Gilbreath TM III, Kukutla P, Yan G, Xu J (2011) Dynamic Gut Microbiome across Life History of the Malaria Mosquito *Anopheles gambiae* in Kenya. PLoS ONE 6(9): e24767. doi:10.1371/journal.pone.0024767.

Cirimotich CM, Dong Y, Clayton AM, Sandiford SL, Souza-Neto JA, Mulenga M, Dimopoulos G. (2011) Natural Microbe-Mediated Refractoriness to Plasmodium Infection in *Anopheles gambiae.* Science 332: 855-859.

Joyce JD, Nogueira JR, Bales AA, Pittman KE, Aanderson JR (2011) Interactions Between La Crosse Virus and Bacteria Isolated From the Digestive Tract of *Aedes albopictus* (Diptera: Culicidae) J. Med. Entomol. 48(2): 389-394.
