# Supplementary material for: Serratia odorifera a Midgut Inhabitant of Aedes aegypti Mosquito Enhances Its Susceptibility to Dengue-2 Virus
Source: PLoS One. 2012 Jul 27;7(7):e40401. doi: 10.1371/journal.pone.0040401 (PMC3407224; doi:10.1371/journal.pone.0040401)
Supplement: Table S2 — P40 binding proteins from brush border membrane of Ae. aegypti. (DOC) [file pone.0040401.s003.doc]

**Table S2: P40 binding proteins from brush border membrane of *Ae. aegypti***

| No. | Accession No. | Protein Description | Mol. Mass (kDa) | | Mass values matched |
| --- | --- | --- | --- | --- | --- |
|  |  |  | From Fig. | From Database |  |
| 1 | **ABF18270** | Porin | 38 | 30.696 | 19 |
| 2 | **ABF18314** | Prohibitin | 32 | 29.885 | 12 |

Sequence coverage was over 25% in all samples.
