# Supplementary material for: Serratia odorifera a Midgut Inhabitant of Aedes aegypti Mosquito Enhances Its Susceptibility to Dengue-2 Virus
Source: PLoS One. 2012 Jul 27;7(7):e40401. doi: 10.1371/journal.pone.0040401 (PMC3407224; doi:10.1371/journal.pone.0040401)
Supplement: Table S3 — Dengue-2 virus binding proteins from S. odorifera cell lysate. (DOC) [file pone.0040401.s004.doc]

**Table S3: Dengue-2 virus binding proteins from *S. odorifera* cell lysate.**

| No. | Protein Description | Mol. Mass (kDa) | | Mass values matched |
| --- | --- | --- | --- | --- |
|  |  | From Fig. | From database |  |
| 1 | Ribosomal RNA large subunit methyltransferase Xanthomonas oryzae pv. Oryzae | 29 | 23.437 | 14 |
| 2 | DNA-directed RNA polymerase subunit alpha Enterobacter sp. (strain 638) | 36 | 36.671 | 12 |

Sequence coverage was over 25% in all samples.
