# Supplementary material for: Serratia odorifera a Midgut Inhabitant of Aedes aegypti Mosquito Enhances Its Susceptibility to Dengue-2 Virus
Source: PLoS One. 2012 Jul 27;7(7):e40401. doi: 10.1371/journal.pone.0040401 (PMC3407224; doi:10.1371/journal.pone.0040401)
Supplement: Table S4 — Functional site comparison of prohibitin with other immunity proteins of Aedes aegypti. (DOC) [file pone.0040401.s005.doc]

**Table S4: Functional site comparison of prohibitin with other immunity proteins of *Aedes aegypti.***

| Functional Site | Defensin | Cercopin | PPO1 | Relish | Prohibitin |
| --- | --- | --- | --- | --- | --- |
| N-Arg dibasic convertase, (nardilysine) cleavage site | Absent | Absent | √ | √ | √ |
| Yeast kexin 2 cleavage site | √ | √ | √ | √ | √ |
| Substrate recognition site that interacts with cyclin and thereby increases phosphorylation by cyclin/cdk complexes | Absent | √ | √ | √ | √ |
| FHA domain interaction motif 1 | √ | Absent | √ | √ | √ |
| Class III PDZ domains binding motif | √ | √ | √ | √ | √ |
| PP1c (protein phosphatase 1 catalytic subunit)- binding motif | Absent | Absent | Absent | Absent | √ |
| Src-family Src Homology 2 (SH2) domains binding motif. | Absent | Absent | Absent | √ | √ |
| Motif recognized by those SH3 domains with a non-canonical class I recognition specificity | √ | Absent | √ | √ | √ |
| Major TRAF2-binding consensus motif. | Absent | Absent | √ | √ | √ |
| CK1 phosphorylation site | Absent | Absent | √ | √ | √ |
| CK2 phosphorylation site | Absent | Absent | √ | √ | √ |
| Glycosaminoglycan attachment site | √ | Absent | √ | √ | √ |
| Generic motif for Nglycosylation. | Absent | Absent | √ | √ | √ |
| PKA phosphorylation site | √ | Absent | √ | √ | √ |
| Site phosphorylated by the Polo-like-kinase | Absent | Absent | √ | √ | √ |
| Tyrosine-based sorting signal responsible for the interaction with mu subunit of AP (Adaptor Protein) complex | Absent | Absent | √ | Absent | √ |
